# Factors affecting the survival of patients with oesophageal carcinoma under radiotherapy in the north of Iran

**DOI:** 10.1054/bjoc.2001.2153

**Published:** 2001-11

**Authors:** K O Hajian-Tilaki

**Affiliations:** Department of Social Medicine and Health, Faculty of Medicine, Babol University of Medical Sciences, Babol, Iran

**Keywords:** oesophageal cancer, survival rate, prognostic factors, radiotherapy

## Abstract

Factors relevant to the survival of patients with oesophageal cancer under radiotherapy have been studied in northern Iran where its incidence is high. We conducted an analytical study using a historical cohort and information from the medical charts of patients with oesophageal cancer. Out of 523 patients referred to the Shahid Rajaii radiotherapy centre in Babolsar from 1992 to 1996, we followed 230 patients for whom an address was available in 1998. The frequency of prognostic factors among those not contacted was very similar to those included in the study. The data were analysed using survival analysis by the nonparametric method of Kaplan Meier and the Cox regression model to determine risk ratios (RR) of prognostic factors. Survival rates were 42% at 1 year, 21% at 2 years, and 8% at 5 years after diagnosis. Patients aged 50–64 were found to have poorer survival compared with those less than 50 (RR = 1.73, *P* = 0.03); the risk ratio for ages f = 65 was 1.88 (*P* = 0.03). Females had significantly better survival than males (RR = 0.71, *P* = 0.02). For each 100 rads dose of radiotherapy, the risk ratio was significantly decreased by 1% (RR = 0.99, *P* = 0.05); for each session of radiotherapy, the risk ratio was significantly decreased by 4% (RR = 0.96, *P* = 0.0001); for each square centimetre size of surface under radiotherapy, the risk ratio significantly increased (RR = 1.002, *P* = 0.04). We did not observe a significant difference on survival by histology, anatomical location of tumours, or type of treatment (*P* > 0.05). Prognosis is extremely poor. © 2001 Cancer Research Campaign http://www.bjcancer.com


*British Journal of Cancer* (2001) **85**(11), 1671–1674
© 2001 Cancer Research Campaign
doi: 10.1054/ bjoc.2001.2153, available online at http://www.idealibrary.com on IDEAL

http://www.bjcancer.com

### Factors affecting the survival of patients with oesophageal carcinoma under radiotherapy in the north of Iran

**KO Hajian-Tilaki**

Department of Social Medicine and Health, Faculty of Medicine, Babol University of Medical Sciences, Babol, Iran

**Summary** Factors relevant to the survival of patients with oesophageal cancer under radiotherapy have been studied in northern Iran where its incidence is high. We conducted an analytical study using a historical cohort and information from the medical charts of patients with oesophageal cancer. Out of 523 patients referred to the Shahid Rajaii radiotherapy centre in Babolsar from 1992 to 1996, we followed 230 patients for whom an address was available in 1998. The frequency of prognostic factors among those not contacted was very similar to those included in the study. The data were analysed using survival analysis by the nonparametric method of Kaplan Meier and the Cox regression model to determine risk ratios (RR) of prognostic factors. Survival rates were 42% at 1 year, 21% at 2 years, and 8% at 5 years after diagnosis. Patients aged 50–64 were found to have poorer survival compared with those less than 50 (RR = 1.73, $P = 0.03$); the risk ratio for ages ⩾ = 65 was 1.88 ($P = 0.03$). Females had significantly better survival than males (RR = 0.71, $P = 0.02$). For each 100 rads dose of radiotherapy, the risk ratio was significantly decreased by 1% (RR = 0.99, $P = 0.05$); for each session of radiotherapy, the risk ratio was significantly decreased by 4% (RR = 0.96, $P = 0.0001$); for each square centimetre size of surface under radiotherapy, the risk ratio significantly increased (RR = 1.002, $P = 0.04$). We did not observe a significant difference on survival by histology, anatomical location of tumours, or type of treatment ($P > 0.05$). Prognosis is extremely poor. © 2001 Cancer Research Campaign http://www.bjcancer.com

**Keywords**: oesophageal cancer; survival rate; prognostic factors; radiotherapy

The north of Iran lies within the central Asian belt of oesophageal carcinoma where the incidence is much higher than in most western populations (Daily et al, 1996). Oesophageal carcinoma is a rapidly progressing disease with a poor prognosis; the median survival is less than 10 months and the 5-year survival rate is less than 10% (Earlam et al, 1980a; Muller et al, 1990; Gaspar et al, 1997).

Whichever therapeutic regimen is followed, the overall results for treatment of oesophageal cancer are poor. There is no agreement on the best way to manage patients with this disease. Although, in recent decades, by improving of surgical techniques and special care after surgery, the operational mortality has decreased dramatically, however, the long term survival has not improved very much (Earlam et al, 1980b). Since the effect of surgery, radiotherapy or chemotherapy alone on survival was poor, the combined use of radiotherapy and chemotherapy simultaneously or surgery with pre- or postoperative radiotherapy had been suggested (Daily, 1996; Earlam et al, 1980a,b; Nicks et al, 1973) but their effect on the survival is still controversial. The efficacy of the combined modalities of radiotherapy with surgery or with chemotherapy on the survival rate have been confirmed in several observational studies (Parker et al, 1976; Lieberman et al, 1995; Daily et al, 1996), but clinical trials did not consistently confirm their effectiveness (Mei et al, 1989; Iizuka et al, 1988).

Among the various indications of survival, the stage of malignant development has been most documented (Sugimachi et al, 1983). However, the influence of the various possible prognostic factors is complex. Survival may depend on several demographic, clinic, pathologic, and tumour-host related variables (Sugimachi et al, 1986). It is important that our knowledge of the prognostic factors for such a fatal neoplasm be updated using data from different areas in a multivariate statistical model. Such updated information on the influence of pretreatment and treatment factors on survival would advance the current stage of knowledge concerning the management of oesophageal patients and hopefully increase the time free of disease.

The Babolsar Shahid Rajaii radiotherapy centre in the north of Iran, has contributed to the therapeutic management of patients with cancer of the oesophagus during the last 25 years, but no information on survival or prognostic factors has been reported from this area. This study aimed to study the 5-year survival of such patients and to identify important prognostic factors.

### METHODS AND MATERIALS

A total of 523 patients were diagnosed with oesophageal carcinoma at the radiotherapy centre of Babolsar Shahid Rajaii between 1992 and 1996. The medical records of all these patients were examined and information collected on potential prognostic factors such as age, sex, area of residence, the anatomical location of tumour, date of diagnosis, histologic type, type of treatment, dose and frequency of radiotherapy, and size of treatment area.

We were able to follow 230 patients for whom a postal address was available in 1998–1999. Survival data, date of death or date last known to be alive were obtained by writing to the families of patients.

*Received 21 March 2001*
*Revised 8 June 2001*
*Accepted 18 September 2001*

*Correspondence to:* KO Hajian-Tilaki

In the analysis, we used the nonparametric Kaplan Meier method and the Cox regression model to study the effect of prognostic factors on survival. In each univariate and multivariate model, we estimated the risk ratio (RR) and its 95% confidence limits.

### RESULTS

The mean age of the 230 patients in the study was 62( + 11) years and modal age group 60–69 years; 59% of the patients were male. The mean dose of radiotherapy was 4500 cGy (rads); the mean number of sessions (frequency) 22; and the mean size of treatment area 148 $cm^2$.

The survival rates (Table 1; Figure 1) were 42% at 1 year after diagnosis, 21% at 2 years, 11% at 3 years and 8% at 5 years. Patients under the age of 50 years survived longer than older patients. Table 2 shows that patients aged 50–64 and over 65 years have risk ratios of 1.73 ($P$ = 0.03) and 1.88 ($P$ = 0.02) compared with patients who were under the age of 50. Table 2 and Figure 2 also show that females had significantly better survival than males (RR = 0.71, $P$ = 0.02) and that there was no difference in risk for patients from rural area compared with those who were urban residents. The risk ratio represents with location of the tumour in the middle and lower third of the oesophagus in comparison with the upper third were 0.88 and 0.74 but the survival times were not significantly lower than patients with tumour located in the upper third of the oesophagus. No effect of histological type was observed. There was a tendency for the combined modality of radiotherapy and chemotherapy to show better survival than radiotherapy alone ($P$ = 0.74) but again this was not significant. However, by increasing the of dose of radiotherapy by 100 cGY, the risk was decreased by 1% (RR = 0.99, $P$ = 0.05) and for each session of radiotherapy, the risk decreased by 4% (RR = 0.96, $P$ = 0.0001).

In multivariate analysis using stepwise Cox regression model, only the effect of age and of dose and frequency of radiotherapy remained significant.

**Table 1** The proportion of patients with oesophageal carcinoma who survive the specified number of months from time of diagnosis with standard errors (SE) by age group

| Time (month) | Age groups | | | | | | | |
|---|---|---|---|---|---|---|---|---|
| | < 50 years | | 50–64 years | | > 65 years | | Total | |
| | Survival rate | SE | Survival rate | SE | Survival rate | SE | Survival rate | SE |
| 1 | 0.96 | 0.03 | 0.97 | 0.02 | 0.96 | 0.02 | 0.96 | 0.01 |
| 3 | 0.85 | 0.06 | 0.89 | 0.03 | 0.84 | 0.03 | 0.87 | 0.02 |
| 6 | 0.71 | 0.08 | 0.66 | 0.05 | 0.64 | 0.04 | 0.65 | 0.03 |
| 9 | 0.50 | 0.09 | 0.50 | 0.05 | 0.54 | 0.05 | 0.52 | 0.03 |
| 12 | 0.50 | 0.09 | 0.41 | 0.05 | 0.42 | 0.04 | 0.42 | 0.03 |
| 18 | 0.50 | 0.09 | 0.24 | 0.05 | 0.24 | 0.04 | 0.26 | 0.03 |
| 24 | 0.39 | 0.09 | 0.19 | 0.05 | 0.17 | 0.03 | 0.21 | 0.03 |
| 30 | 0.36 | 0.09 | 0.15 | 0.04 | 0.11 | 0.03 | 0.15 | 0.02 |
| 36 | 0.32 | 0.09 | 0.11 | 0.03 | 0.06 | 0.02 | 0.11 | 0.02 |
| 48 | 0.27 | 0.09 | 0.04 | 0.03 | 0.04 | 0.02 | 0.08 | 0.02 |
| 60 | 0.27 | 0.09 | 0.04 | 0.03 | 0.04 | 0.02 | 0.08 | 0.02 |

**Table 2** Coefficients of the Cox regression model with standard errors (SE) and the risk ratios of prognostic factors with 95% confidence intervals (CI) and *P*-values from the univariate analysis

| Prognostic factors | Coefficient (B) | SE (B) | Risk Ratio (RR) | 95% CI RR | *P*-value |
|---|---|---|---|---|---|
| Age groups | | | | | |
| < 50 years | – | – | 1 | – | – |
| 50–64 years | 0.55 | 0.25 | 1.73 | 1.06–2.82 | 0.03 |
| > 65 years | 0.63 | 0.24 | 1.88 | 1.16–3.04 | 0.01 |
| Sex | | | | 0.53–0.94 | 0.02 |
| female vs male | –0.34 | 0.14 | 0.71 | | |
| Area of residence rural vs urban | 0.009 | 0.14 | 1.01 | 0.76–1.33 | 0.94 |
| Anatomical location | | | | | |
| upper third | – | – | 1 | – | – |
| medial third | –0.19 | 0.23 | 0.83 | 0.53–1.29 | 0.41 |
| lower third | –0.29 | 0.23 | 0.74 | 0.47–1.17 | 0.20 |
| Histologic | | | | | |
| SCC vs adeno | 0.11 | 0.25 | 1.11 | 0.68–1.83 | 0.66 |
| Radiotherapy and chemotherapy vs | | | | | |
| radiotherapy alone | 0.05 | 0.14 | 1.05 | 0.79–1.39 | 0.74 |
| Palliative vs radical | 0.13 | 0.17 | 1.13 | 0.81–1.59 | 0.46 |
| Dose (100 rads) | –0.008 | 0.004 | 0.99 | 0.98–1.0 | 0.05 |
| Size of surface ($cm^2$) | 0.002 | 0.001 | 1.002 | 1.001–1.004 | 0.04 |
| Frequency | –0.035 | 0.008 | 0.96 | 0.94–0.98 | 0.001 |

SCC: squamous cell carcinoma.

*© 2001 Cancer Research Campaign*

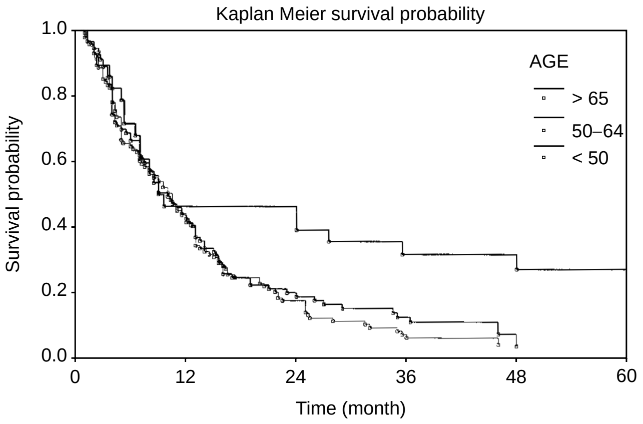

**Figure 1.** Survival of patients with oesophageal carcinoma in different age groups

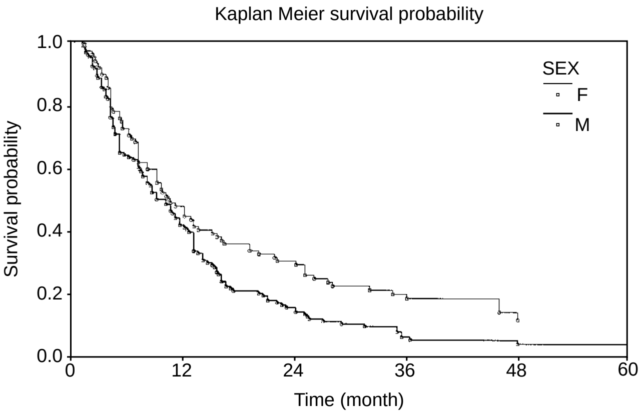

**Figure 2.** Survival of patients with oesophageal carcinoma in males and females

### DISCUSSION

Our results show that overall survival rate of patients with oesophageal carcinoma under radiotherapy at 1 and 5 years were 42% and 8% respectively. These results are consistent with those had been reported in the western counties that the 5-year survival was less than 10% (Daily et al, 1996; Earlam et al, 1980a,b).

The greatest improvements the treatment of patients of the oesophagus have occurred in China and Japan in the last decade. For example, Chen et al (1996) in China reported that 1-year and 5-year survival of patients under radiotherapy were 64% and 23% respectively. This improvement of survival rates, may be due to the screening program and the treatment of patients at an early stage of the disease.

In this study, we found that age and gender, dose and frequency of radiotherapy and the size of surface under radiotherapy are the important prognostic factors for survival. Several previous studies have also found longer survival times for women (Van Andel, 1979; Kinoshita et al, 1982; Shimada et al, 1999). Thus, the recurrence or progression of oesophagus carcinoma may relate to a hormonal factor such as testosterone or androgen. Other studies, however, have not found significant differences in survival between men and women (Bluett et al, 1987; Koth et al, 1999; Malhair et al, 1998; Jeremic et al, 1998).

We observed that patients with age < 50 years had better long-term survival than those aged > = 50 years and that the 5-year survival at ages < 50 was 27% compared with 4% at ages > = 50; the risk is greater for older patients, roughly 88% of the first 5 years after diagnosis. Lund et al (1989) reported that patients at younger ages (< = 55 years) had slightly poorer survival than older patients (55–64); but after this age, there is an inverse relationship with survival. In other retrospective studies, it was also demonstrated that patients aged 65 and over had poorer survival than younger patients (Laumous et al, 1983; Petrequin et al, 1977; Stevin et al, 1989). In contrast, Koth et al (1999) and Giuli and Gignoux (1980) did not observe an effect of age on survival; while, Oliver et al (1989) in a retrospective study of 495 patients with oesophageal cancer, an inverse association of survival time with age was observed.

According to our results, patients with squamous cell carcinoma had slightly poorer survival compared with adenocarcinoma in the first year after diagnosis, but the differences between histological types of tumour were not statistically significant. This result is also consistent with those found in other studies (Foratiere et al, 1997; Shwartz et al, 1999; Schahemberk et al, 1987; Lieberman et al, 1995).

Our findings indicated that patients with tumours located in the medial and lower third of oesophagus compared with the upper third, tended to have slightly better survival. However, again the differences were not statistically significant. Cederquist et al (1978) also reported that there is no association between the anatomical location of tumours and survival but Stevin et al (1989) found that the 5-year survival rate for patients under radiotherapy with anatomical location of tumour in the upper third was 15.6% versus 3.2% for lower third.

With regard treatment, we found that the dose and frequency of radiotherapy and the size of surface treated appear to influence survival. From published studies, the efficacy of radiotherapy depends on the site and stage of the tumour and on histological type. In particular, Giuli et al (1980) reported that for SCC of the upper third of the oesophagus, radiotherapy had apparently better results than resection. In general, radiotherapy is used only for those patients who are unfit for surgery due to the their medical condition, older age, or the presence of a tumour with nodal involvement or invasive metastasis. There is no controlled randomized trial of radiotherapy versus surgery for squamous cell carcinoma that evaluates the efficacy of radiotherapy compared with surgery. Published data indicate that the biological effect of radiation depends on the dose and frequency and the size of surface under radiotherapy (Vincent et al, 1993) which are consistent with those we found in this study.

Although, many retrospective studies appear to confirm the superiority of adjuvant radiotherapy and chemotherapy over radiotherapy alone in improving long-term survival (Daily et al, 1996; Lieberman et al, 1995). In our observational study, we did not find a significant difference between the combined modality of radiotherapy and chemotherapy versus radiotherapy alone. In observational studies, the comparison of different treatments may be confounded by clinical indications for which it is difficult to control. However, in a randomized clinical trial, Herskovic et al (1992) indicated that chemotherapy in combination with radiotherapy yields significantly better long-term survival than radiotherapy alone (2-year survival 38% vs 10%) but that the frequency of several life threatening side effects was higher with combined therapy. Arauijo et al (1991) also reported that radiotherapy and chemotherapy with 5-fluoirourcil, Miltomycin C and Bleomycin slightly improved survival over that achievable with radiotherapy

*© 2001 Cancer Research Campaign* 

alone. Also, Onsuto and et al (1995) examined the effect of combined modality therapy on advanced oesophagus carcinoma and concluded that combined modality is the optimal way for the management of advanced cases.

A limitation of the present study is that we do not have data on the staging and on spread to lymph nodes, on the size of tumour or the depth of penetration and vascular invasion. The effect on prognosis of these factors is well documented (Sugimachi et al, 1983; Kakegaw et al, 1995).

Another potential limitation of this study is the lack of follow-up of some patients that work treated at the Babolsar centre. Out of 523 patients who were treated during the period of 1992–1996, we were able to follow up 230 patients based on the availability of their address in 1998. However, in our analysis, we did not find any difference in the profile of prognostic factors between those we followed up and those we did not and then our results can be generalized for all patients who were treated within the period of 1992–1996. However, if those who had no adequate address information were poorer that those who were traced, they may have died sooner and so lowered the overall survival truth.

Our results indicate that survival depends on age, sex, dose and frequency of radiotherapy and the size of surface under radiotherapy but the overall prognosis remains poor. For a clearer indication of the optimal way to manage patients with oesophageal carcinoma, a multi-centred randomized clinical trial of various combined modalities with adequate sample sizes is still necessary.

### REFERENCES

Arauijo GM, Souhami L and Gil RA (1991) A randomized trial comparing radiotherapy versus concomitant radiation therapy and chemotherapy in carcinoma of thoracic oesophagus. *Cancer* **67**: 2258–2261

Bluett MK and Sawyer JL (1987) Oesophageal carcinoma improvement quality of survival with resection. *Am J Surg* **53**: 126–132

Cederquist C, Nielsen J and Berthesen A (1978) Cancer of the oesophagus: I-1002 cases, survey and survival, *Acta Chir Achad* **144**: 227–231

Chen D, Yang Z and Gu X (1996) Radiation therapy of oesophagus carcinoma. *Chung-Hua-Chung-Liu-Tsa-Chih* **18**: 195–198

Daily JM, Karnel LH and Menck HR (1996) National cancer data base reports on oesophageal carcinoma. *Cancer* **78**: 1820–1828

Earlam R and Cunha-Melo JR (1980a) Oesophageal squamous cell carcinoma: I. A critical review of surgery. *Br J Surg* **67**: 381–390

Earlam R and Cunha-Melo JR (1980b) Oesophageal squamous cell carcinoma: II. A critical review of radiotherapy *Br J Surg* **67**: 457–461

Foratiere AA, Heitmiller RF and Lee DJ (1997) Intensive chemoradiation followed by esophagectomy for squamous cell and adenocarcinoma of the oesophagus. *Cancer J Sci Am* **3**: 144–152

Gaspar LE, Qian C, Kocha WI, Coia LR, Herskovic A and Graham M (1997) A phase I/II study of external beam radiation, brachy therapy and concurrent chemotherapy in localized cancer of oesophagus. *Int J Radiat Oncol Biol Phys* **37**: 593–599

Giuli R and Gignoux M (1980) Treatment of carcinoma of oesophagus. *Ann Surg* **192**: 44–52

Herskovic A and Martz K and Al-sarraf MA (1992) Combined chemotherapy and radiotherapy compared with radiotherapy alone in patients with cancer of oesophagus. *N Engl J Med* **326**: 1953–1958

Iizuka T (1988) Preoperative radiotherapy for oesophageal carcinoma: Randomized clinical evaluation trial in eight institutes. *Chest* **93**: 1054

Jeremic B, Shibamoto Y, Acimmovic L, Matovie Z and Milicic B (1998) Accelerated hyperfractionated radiation therapy and concurrent 5-Fluorouracil/cisplatin chemotherapy for locoregional squamous cell carcinoma of the thoracic oesophagus. *Int J Radiol Oncol Biol Phys* **40**: 1061–1066

Kakegaw I and Yamanan H (1995) Prospective prognostic factor for carcinoma of the thoracic oesophagus. *Gan Io Kagaka Ryoho* **22**: 575–590

Kinoshita Y, Endo M and Nakayama K (1982) Clinical evaluation of ten-year survival cases after operation for upper and mid thoracic oesophageal cancer. *Int J Surg* **67**: 153–161

Koth P, Honore P, Gielen JL, Degauque C, Legrand M and Jacqwuet N (1999) Analysis of factors influencing long term survival after surgical resection for oesophageal squamous cell carcinoma. *Acta Chir Belg* **99**: 113–118

Laumous B, Paul JL and Lygidakis NJ (1983) Results of the surgical treatment of oesophagus. *Surg Gynec Obstet* **156**: 735–740

Lieberman MD, Shriver CD, Bleckner S and Blunt M (1995) Carcinoma of oesophagus: prognostic significant of histologic type. *J Thorac Cardiovase Surg* **109**: 105–108

Lund O, Hasenkam JM, Agord MI and Kimose MH (1989) Time related changes in characteristic of prognostic significant of oesophagus and cardia. *Br J Surg* **76**: 1301–1307

Malhair JP, Lozac HP and Simon H (1998) Split-course concomitant radiochemotherapy plus surgery vs surgery alone in squamous cell carcinoma of the oesophagus. *Bull Cancer* **84**: 357–367

Mei W, Xian-Zhi G and Yian W (1989) Randomized clinical trial of the combination of preoperative irradiation and surgery in the treatment of oesophageal carcinoma: Report of 206 patients. *Int J Radiol Oncol Biol Phys* **16**: 325–327

Muller JM, Erasmi H and Stelzner M (1990) Surgical therapy of oesophageal carcinoma. *Br J Surg* **77**: 845–847

Nicks R, Green D and Mc Catchie G (1973) A clinical study of some factor influencing survival in cancer of oesophagus: A survey often year experience. *Aust N Z J Surg* **43**: 3–13

Oliver SE, Rohertson CS and Logan RFA (1989) Oesophageal carcinoma improvement quality of survival with resection. *Am J Surg* **79**: 1321–1325

Onsuto A, Yoshida S, Boku N and Fugil I (1995) Concurrent chemotherapy and radiation therapy for locally advanced carcinoma of the oesophagus. *Jpn J Clin Oncol* **25**: 2161–2166

Parker EF and Gregorie HB (1976) Oesophagectomy without thoracotomy: Carcinoma of oesophagus long term results: *JAMA* **35**: 1018

Petrequin P, Huguier M, Lacaine F and Houry S (1977) Surgical treated oesophageal cancer: predicted model of survival. *Gastroenterol Clin Biol* **21**: 12–16

Schahemberk ME, Obetrtop H and Mud HJ (1987) Survival after resection for carcinoma of oesophagus. *Br J Surg* **74**: 165–168

Schwartz, Shires, Spencer, Daly, Fisher and Galloway (1999) *Principles of surgery*, 7th edn, P1081. McGraw Hill Company: New York

Shimada Y, Imamura M, Watanaba G, Uchida H, Harada H, Makino T and Kano M (1999) Prognostic factors of oesophageal squamous cell carcinoma from the perspective of molecular biology. *Br J Cancer* **80**: 1281–1288

Stevin NI and Stout R (1989) Carcinoma of oesophagus: A review of 108 cases treatment by radical radiotherapy. *Clin Radiol* **40**: 200–203

Sugimachi K, Inokuchi K and Kuvano H (1983) Pattern of recurrence after arative resection for carcinoma of thoracic of oesophagus. *Surg Gynecol Obstet* **157**: 537–540

Sugimachi K, Matsura H and Kall M (1986) Prognostic factor of oesophageal carcinoma: univariate and multivariate analysis. *J Surg Oncol* **31**: 108–112

Van Andel JG, Dees GJ and Dijikbuis CM (1979) Carcinoma of oesophagus. *Ann Surg* **190**: 684–689

Vincent T, Devita Jr, Sarmuel H, Steven A and Rosenberg (1993) *Principles and practice of oncology*, 4th edn. pp 776–813. Lippincott Company: Philadelphia

*© 2001 Cancer Research Campaign*
